# Retraction: Low temperature synthesis of superparamagnetic iron oxide (Fe_3_O_4_) nanoparticles and their ROS mediated inhibition of biofilm formed by food-associated bacteria

**DOI:** 10.3389/fmicb.2026.1812892

**Published:** 2026-02-26

**Authors:** Frontiers Editorial Office

The journal retracts the 05 November 2018 article cited above. Following publication, concerns were raised regarding the integrity of the images in the published figures. Image duplication concerns were identified in Figure 7B. The authors failed to provide a satisfactory explanation during the investigation, which was conducted in accordance with Frontiers' policies. As a result, the data and conclusions of the article have been deemed unreliable and the article has been retracted.

This retraction was approved by the Chief Editors of Frontiers in Microbiology and the Chief Executive Editor of Frontiers. The authors do not agree to this retraction.

Frontiers would like to thank the users on PubPeer for bringing the published article to our attention.

